# Carbon dots hybrid for dual fluorescent detection of microRNA-21 integrated bioimaging of MCF-7 using a microfluidic platform

**DOI:** 10.1186/s12951-022-01274-3

**Published:** 2022-02-08

**Authors:** Somayeh Mohammadi, Abdollah Salimi, Zohreh Hoseinkhani, Foad Ghasemi, Kamran Mansouri

**Affiliations:** 1 Department of Chemistry, University of Kurdistan, 66177-15175 Sanandaj, Iran; 2 Research Center for Nanotechnology, University of Kurdistan, 66177-15175 Sanandaj, Iran; 3 Medical Biology Research Center, Kermanshah University of Medical Sciences, Kermanshah, Iran; 4 Nanoscale Physics Device Lab (NPDL), Department of Physics, University of Kurdistan, 66177-15275 Sanandaj, Iran

**Keywords:** Ratiometric fluorescence, Microfluidic, MicroRNA-21, Dual emissive assay, Carbon dots, Cell imaging

## Abstract

**Background:**

MicroRNAs have short sequences of 20 ~ 25-nucleotides which are similar among family members and play crucial regulatory roles in numerous biological processes, such as in cell development, metabolism, proliferation, differentiation, and apoptosis.

**Results:**

We reported a strategy for the construction of a dual-emission fluorescent sensor using carbon dots (CDs) and confirmed their applications for ratiometric microRNA-21 sensing and bioimaging of cancer cells in a microfluidic device. The composition of blue CDs (B-CDs) and yellow CDs (Y-CDs) depicts dual-emission behavior which is centered at 409 and 543 nm under an excitation wavelength of 360 nm. With increasing microRNA-21 concentration, the robust and specific binding of DNA probe functionalized B-CDs to complementary microRNA-21 target induced perturbations of probe structure and led to changing fluorescence intensity in both wavelengths. Consequently, the ratio of turn-on signal to turn-off signal is greatly altered. With monitoring of the inherent ratiometric fluorescence variation (ΔF_540nm_/ΔF_410nm_), as-prepared BY-CDs were established as an efficient platform for ratiometric fluorescent microRNA-21 sensing, with a wide linear range of 0.15 fM to 2.46 pM and a detection limit of 50 aM.

**Conclusions:**

Furthermore, the proposed assay was applied for detecting microRNA-21 in dilute human serum samples with satisfactory recovery and also in MCF-7 cell lines in the range 3000 to 45,000 (cell mL^−1^) with a detection limit (3 cells in 10 μL), demonstrating the potential of the assay for clinic diagnosis of microRNA-associated disease. More importantly, the images revealed that MCF-7 cells well labeled with BY-CDs could exhibit the applicability of the proposed microfluidic system as an effective cell trapping device in bioimaging.

**Graphical Abstract:**

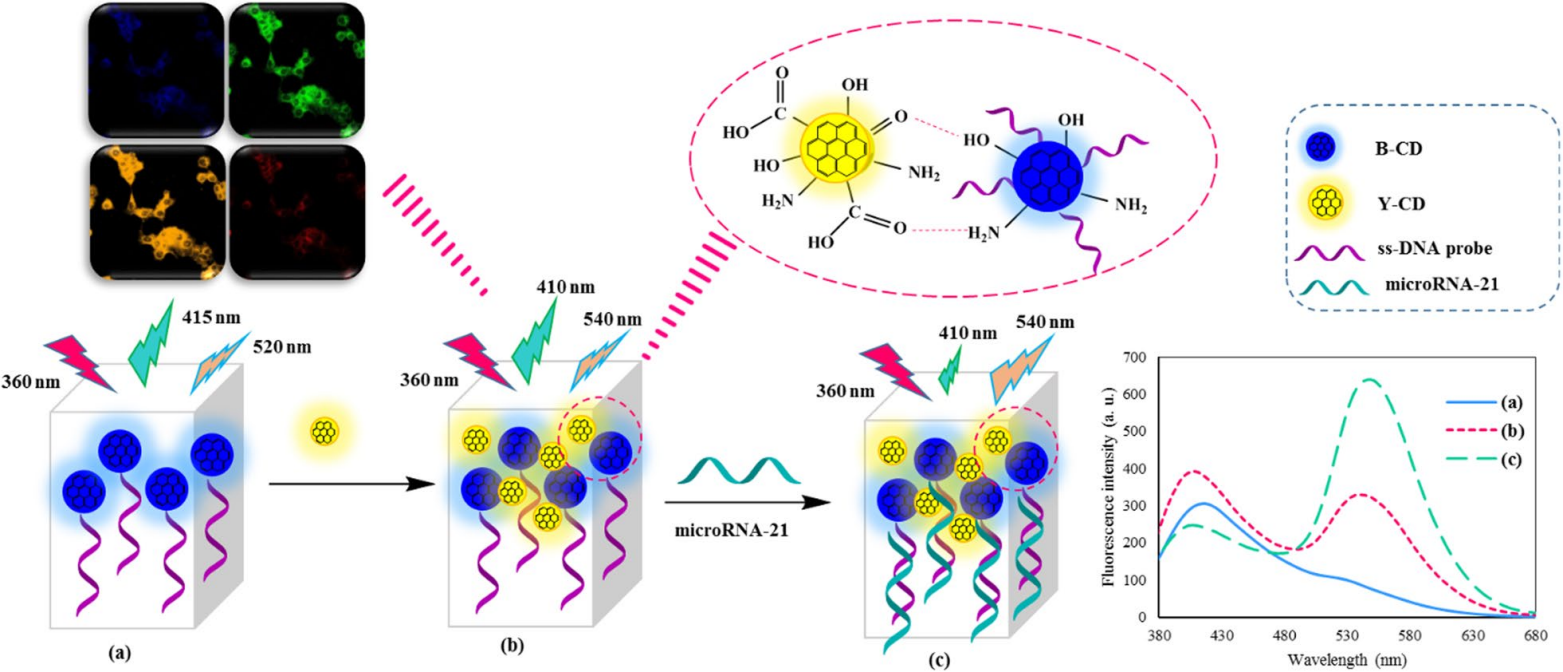

**Supplementary Information:**

The online version contains supplementary material available at 10.1186/s12951-022-01274-3.

## Background

MicroRNAs, a class of endogenous single-stranded non-coding RNAs, have regulated gene activity in cell development [1], metabolism [2], proliferation [3], differentiation [4], and apoptosis [5]. There is a recognizable connection among dysregulation of microRNA expressions and numerous genetic disorders, such as diabetes [6], neurological diseases [5], viral infections [7], stroke-induced tissue injury [8], and cancers [9]. For instance, microRNA-21 is a robust oncogenic microRNA, not only does its overexpression contribute to a remarkable role in the differentiation, development, and apoptosis of normal cells and course in the characterization of cancer phenotype, but it also can be utilized as a diagnosis, staging, progression and prognosis biomarker for different human malignancies [10, 11]. Determination of microRNAs has encountered numerous challenging tasks owing to their short and extreme similarity of sequences, low abundance in whole RNA samples, and extensive dynamic range [12].

The conventional microRNAs detection systems, including polymerase-chain-reaction (PCR) [13], northern blotting [14], and microarray [15], have been broadly employed as reliable and gold-standard procedures for microRNA profiling but these approaches suffer from the restrictions of low sensitivity, complicated operation, time-consuming methodology, and costly equipment, which confine their practical usage [16, 17].

Developing effective approaches for sensitive microRNAs determination in clinical diagnosis is worth striving for. Ratiometric sensing methods, in which the detection relies on the intensity ratio of emission wavelengths instead of one emission wavelength as signal outputs, have become an ideal approach to quantify target analytes. The intensity ratio-based assays can enable enhanced analytical performance because the ratio of two signals is not affected by the interference factors, such as path length, background light, and scattering by self-calibration [18]. Numerous ratiometric electrochemistry [19, 20], electrochemiluminescence [21], chemiluminescence [22], surface-enhanced Raman spectroscopy [23], and fluorescence [24–26] methods have been reported for extremely accurate and sensitive quantification of microRNA. Among them, ratiometric fluorescent procedures have become increasingly widespread in analytical usage because of their intrinsic benefits such as quick response, high sensitivity, and relatively inexpensive. The detection limit in the pM range was not sufficient to be exploited for the determination of microRNAs in real samples [26, 27]. Therefore, the construction of highly sensitive ratiometric fluorescence methods for microRNAs detection to make them practical in clinical diagnosis is significant [26].

Recently, with the remarkable attainments of nanotechnology, nanomaterial-based biosensors have been significantly expanded owing to the distinctive electronic, optical, and catalytic features of the nanomaterials, which enable the signal amplification to attain high sensitivity [28, 29]. Recently, numerous functional nanomaterials, such as graphene [30], CDs [31], MoS_2_ nanosheets [32], MnO_2_ [31], Conjugated polyelectrolytes [33], and metal–organic framework (MOF) [24], were employed as carriers and quenchers to decrease the background signal and enhance the signal-to-noise ratio [26]. Nanomaterials have contributed to noteworthy enhancements in the design and expansion of ratiometric fluorescence probes, because of the cooperation between nanomaterial and ratiometric fluorescence assay [29].

There have been essential recent attention to the development of nanomaterials with high fluorescent signal for optical imaging and sensing applications. An ideal fluorescent agent should be biocompatible, non-toxic, bright, and photostable against photobleaching. The poor photo-stability, relatively short lifetimes, narrow excitation ranges, and broad emission spectra of fluorescence dyes confines their practical usages in long-term bioimaging. Quantum dots (QDs) exhibit numerous improved optical features, like high brightness and long-term photo-stability, being desirable for biological uses rather than organic dyes. While they have some drawbacks, including the low solubility in water and toxicity restricted their applications in bioimaging [34–36]. CDs, as a member of newly emerging photoluminescent zero-dimensional carbonaceous nanomaterials, offer various improved features, such as convenient synthesis, good water-solubility, adjustable emission spectrum controlled by altering the synthesis conditions, multicolored emissions, and inherent biocompatibility [37, 38]. Therefore, these fluorescent carbon nanomaterials can be appropriate substitutes for fluorescent dyes and semiconductor QDs owing to their unique capability in the field of cell analysis [38].

Biomedical research principally concentrates on the development of effective technologies, like biosensors, image processing, and disease diagnosis [39]. Microfluidic technologies have the potential to develop a capable tool in cellular experiments since it offers distinctive capabilities, like high-throughput potential under various situations, less sample and reagent need, easy construction, and the capability for in vivo and in vitro cellular imaging. Another benefit of microfluidics is the capability to be efficiently combined with other analytical techniques, such as optical detection, electrochemistry, and mass spectrometry. Profited from the progress of microfluidic culture and fluorescence labeling methods, chips integrated with fluorescent devices have converted to a robust tool for cell investigation [11].

Herein, we introduced a new ratiometric nanohybrid fluorescent probe for the detection of microRNA-21 with dual-colored CDs (blue CDs and yellow CDs) as fluorophores. This distinctive combination of Y-CDs and B-CDs supplied two discrete and stable emission signals (409 and 543 nm) under the same excitation wavelength (360 nm). In the presence of microRNA-21, the emission signal in 409 nm was decreased while fluorescence intensity in 543 nm was increased. Great potential for distinguishing between complementary and single-base mismatch microRNA strands has confirmed the great selectivity of the nanosensor for gene mutation assessment. More importantly, the excellent analytical performance of the present assay has been verified in the complex biological medium of cancer cells. The suggested system has been utilized for imaging MCF-7 cancer cells with great reliability and contrast. Moreover, the dual-colored CDs have been assessed as a fluorescent nanoprobe for cellular imaging using a microfluidic system. Taken together, the present procedure enables a great device for microRNA-related biological research.

## Experimental section

### Materials and instruments

All of the DNA, microRNA oligonucleotide sequences were obtained from BIONEER, Global Genomics Partner (http://www.bioneer.com), and purified using HPLC. Their sequences were as follows:

DNA probe: [C6 Amine] TCAACATCAGTCTGATAAGCTA

MicroRNA-21 target: UAGCUUAUCAGACUGAUGUUGA

1 base mismatched microRNA-21: UAGCUUAUCACACUGAUGUUGA

MicroRNA-155 target: UUAAUGCUAAUCGUGAUAGGGGU

1 base mismatched microRNA-155: UUAAUGCUACUCGUGAUAGGGGU

1-Ethyl-3-(3-dimethyl aminopropyl) carbodiimide hydrochloride (EDC), and N-hydroxy succinimide (NHS) were purchased from Abcam Company (Cambridge, UK, http://www.Abcam.com). Dialysis bag (molecular weight cut off = 1000 KDa) was obtained from Sigma-Aldrich (https://www.sigmaaldrich.com). O-phenylenediamine (O–PD), 2-Aminoterephthalic acid, and DMF were ordered from Merck Company (Darmstadt, Germany, http://www.merck.com). The human breast cancer cell line include MCF-7 was acquired from the national cell bank of Iran (Pasteur Institute of Iran, Tehran) and was cultured by confirmed protocols. The TEM images were performed on an H600 TEM (Hitachi, Japan) for specifying of size and shape of prepared CDs. The fluorescence emission spectra were achieved on a Perkin Elmer fluorescence LS55 spectrophotometer (Germany) with the tool settings as follows: λ_ex_ = 360 nm (slit 10 nm), λ_em_ = 370–670 nm (slit 10 nm), PMT detector voltage = 670 V. Laser scanning confocal microscope (Nikon, ECLIPSE, Ti, Japan) was utilized for relative cell imaging.

### Synthesis of blue-emitting carbon dots (B-CDs)

**Scheme 1:**
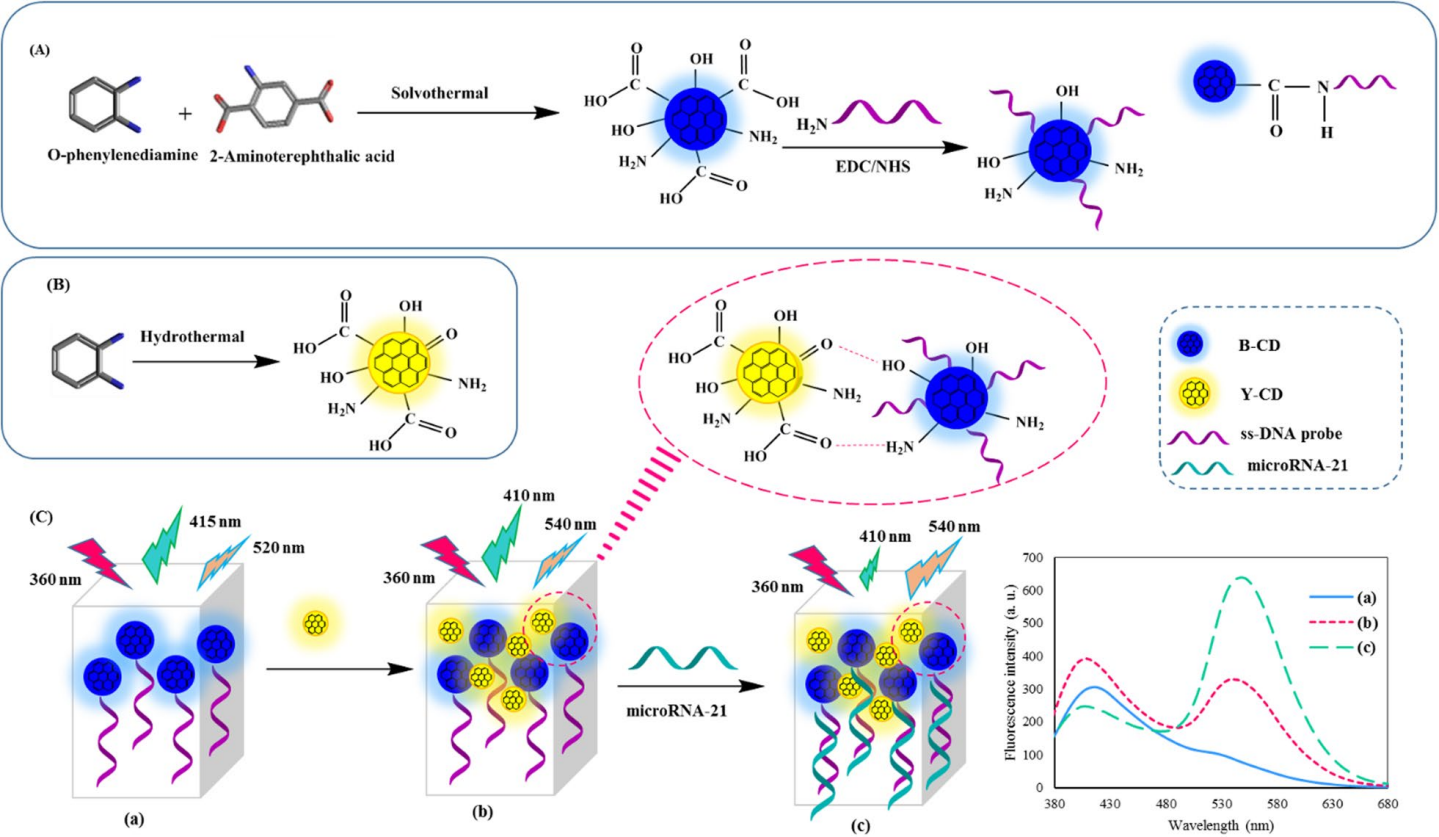
The schematic illustration of the dual-emissive optical method for microRNA-21 detection; synthesis and functionalization of B-CDs (**A**), synthesis of Y-CDs (**B**), and procedure of microRNA-21 detection

B-CD was synthesized using the solvothermal route. The mixture of O-phenylenediamine (OPDA) (50.0 mg, 0.46 mol) and 2-Aminoterephthalic acid (75.0 mg, 0.41 mol) was first dissolved in a 30 mL mixed solvent of DMF and water with a volume ratio of 5/25. Then, the mixed solution was transferred to a Teflon-lined stainless autoclave and heated in an air-circulating oven at 120 °C for 24 h (Scheme 1A). Subsequently, the autoclave was cooled down to room temperature naturally. The orange solution was collected by centrifugation (3500 rpm for 10 min) to remove large particles. Then the upper solution was put into a dialysis membrane (1000 Da, molecular weight cut-off) against deionized water to remove some free molecules. The concentration of purified B-CDs solution was about 1.15 mg·mL^−1^ as measured using the dry weight analysis procedure. The final solution was stored in a refrigerator at 4 °C for further research.

### Synthesis of yellow emitting carbon dots (Y-CDs)

Y-CDs were synthesized according to a previously reported method [40] with proper modification. Briefly, 500 mg of OPDA was dissolved in 50 mL of water (Scheme 1B). Subsequently, the mixed solution was put in a Teflon-lined autoclave and heated at 180 °C for 12 h, then cooled to room temperature. The dark yellow product was centrifuged at 3500 rpm for 5 min to remove solid particles and collect the supernatants. The supernatants were dialyzed against distilled water at 4 °C for 24 h for further purification. The final solution (1.4 mg mL^−1^) was kept at 4 °C for further usage.

### Ratiometric fluorescence detection of microRNA-21

The B-CDs were functionalized with a DNA probe performed through carbodiimide-activated coupling between B-CDs and NH_2_-modified DNA probe. In a typical process, 100 µL ethyl-3-(3-dimethyl aminopropyl) carbodiimide (EDC) (0.01 mol L^−1^) and 100 µL N-hydroxysuccinimide (NHS) (0.01 mol L^−1^) were added to 1.0 mL of the aforementioned CDs (1.15 mg mL^−1^) and kept at room temperature for 1 h. Then, 50 μL DNA probe (1.0 µM) was added to this solution and kept in the refrigerator for 24 h (Scheme 1A). Excessive EDC and NHS were removed by dialysis [41]. The quantitative detection of microRNA-21 using the BY-CDs method was achieved as follows: To construct the dual emission probe, B-CDs and Y-CDs were mixed in 3 different volume ratios (2:1, 1:1, 1:2), and diluted with 270 µL of ultrapure water. Then different microRNA concentrations were added to above sensing system. After incubation for 20 min, the fluorescence signal was recorded in the 370–670 nm emission range under excitation at 360 nm (Scheme 1C). Each measurement was repeated three times.

### Cell culture and cytotoxicity assays

The MCF-7 cells were cultured in DMEM containing 10% (v/v) fetal bovine serum (FBS), 1% (v/v) penicillin at 37 °C in a humid environment of 5% CO_2_. MTT assay was applied to evaluate the cytotoxicity of BY-CDs. MCF-7 cells were cultured for 24 h under the aforementioned situations. After eliminating the culture medium, various amounts of BY-CDs (0, 0.05, 0.1, 0.2, 0.5, 1.0 mg mL^−1^) were added into 96-well plates and were kept in an incubator for another 24 h. After that, the culture medium was separated. Then 200 μL of fresh DMEM containing 10% MTT (20 μL) was added to each well. After 3 h, the supernatant solution was substituted with 100 μL of DMSO to dissolve MTT. Following this, the 96-well was shaken at room temperature for 15 min. Finally, the optical density of each well was recorded at 545/630 nm with a microplate reader. Cell viability (%) was calculated using the following equation:

Cell viability (%) = (OD_treated_/OD_control_) × 100%.

### Imaging of microRNA-21 in fixed MCF-7 cell lines

The cell imaging tests were verified using MCF-7 cells on a confocal laser scanning microscope (CLSM) with a 40 × objective. The imaging potential of BY-CDs fluorescent probe for microRNA detection in cells was evaluated using MCF-7 cancer cells. Briefly, 1 mL of MCF-7 cell suspensions were seeded in 6-well culture dishes at a density of 7 × 10^3^ cells at 37 °C in a humid environment containing 5% CO_2_ for 24 h. Then the medium was substituted by a fresh medium containing 0, 0.5, and 1.0 mg mL^−1^ BY-CDs, the cells have been incubated for 24 h. After washing twice with PBS, the cells were fixed with formaldehyde 4% and then were washed thrice with PBS.

### Construction of the microfluidic device

Firstly, a silicon mold was constructed by the Reactive Ion Etching (RIE) method. Polydimethylsiloxane (PDMS) is the most common substance for microfluidic fabrication. A mixture of PDMS prepolymer and curing agent (10:1) was cast onto the pre-etched silicon mold and cured at 80 °C for 12 h. After patterning of a PDMS substrate by replica molding from a silicon mold, the PDMS replica surface and a glass substrate were activated with reactive oxygen plasma and bonded together, and then permanently sealed to construct channels [42].

## Results and discussion

### Characterization of CDs

**Fig. 1:**
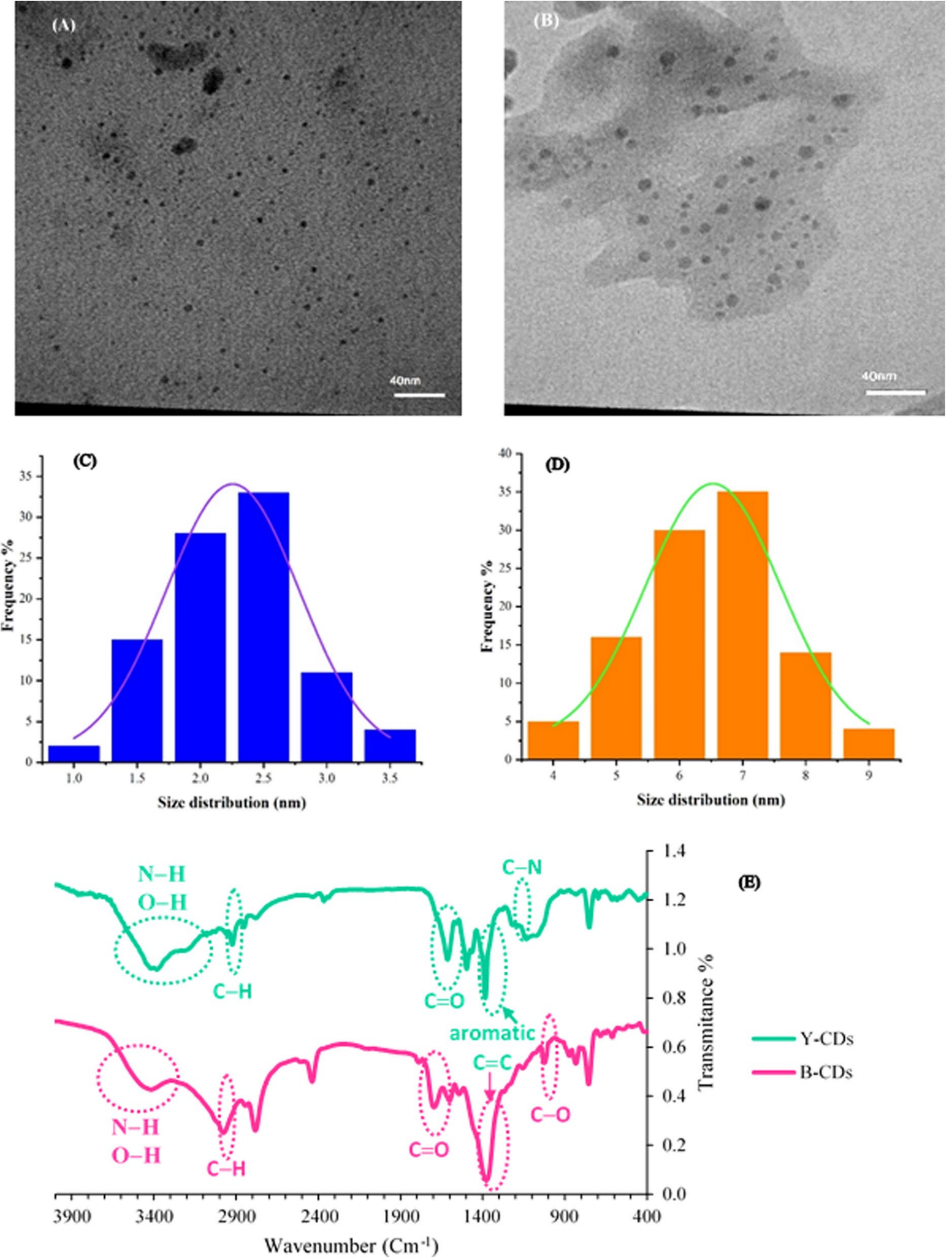
TEM images and the size distribution histograms of B-CDs (**A**, **C**) and Y-CDs (**B**, **D**); The FTIR spectra of Y-CDs and B-CDs (**E**)

In this study, a CDs-based ratiometric fluorescent probe was constructed for the detection of microRNA-21 as a target. The formation of CDs was proved by SEM image, which is displayed in Additional file 1: Fig. S1. TEM images and the size distribution histograms of B-CDs and Y-CDs are displayed in Fig. 1A–D which revealed that the B-CDs and Y-CDs are sphere-shaped and have a mediocre diameter of 2.25 ± 0.94 and 6.47 ± 1.87 nm, respectively. The CDs are further characterized by FTIR. In detail, as shown in Fig. 1E, the FTIR spectra of Y-CDs and B-CDs exhibit a characteristic broadband between 3100 and 3600 cm^−1^, which are ascribed to stretching vibrations of N–H and O–H. The band appears at 2800–2950 cm^−1^ is attributed to stretching vibrations of C–H. The band that is very common to ascribe carboxylic/carbonyl functional groups from CDs, is a band in the range of 1600–1770 cm^−1^ as a piece of evidence from the vibrational stretching of C=O. The peaks at 1027 and 1207 cm^−1^ can be ascribed to the C–O and C–N stretching frequencies, respectively. The vibration band at 1389 cm^−1^ demonstrated the presence of an aromatic C=C group [43]. These results reveal that Y-CDs and B-CDs were comprised of aromatic structures and had amine and carboxyl groups on their surface.

### Optical properties of CDs

**Fig. 2:**
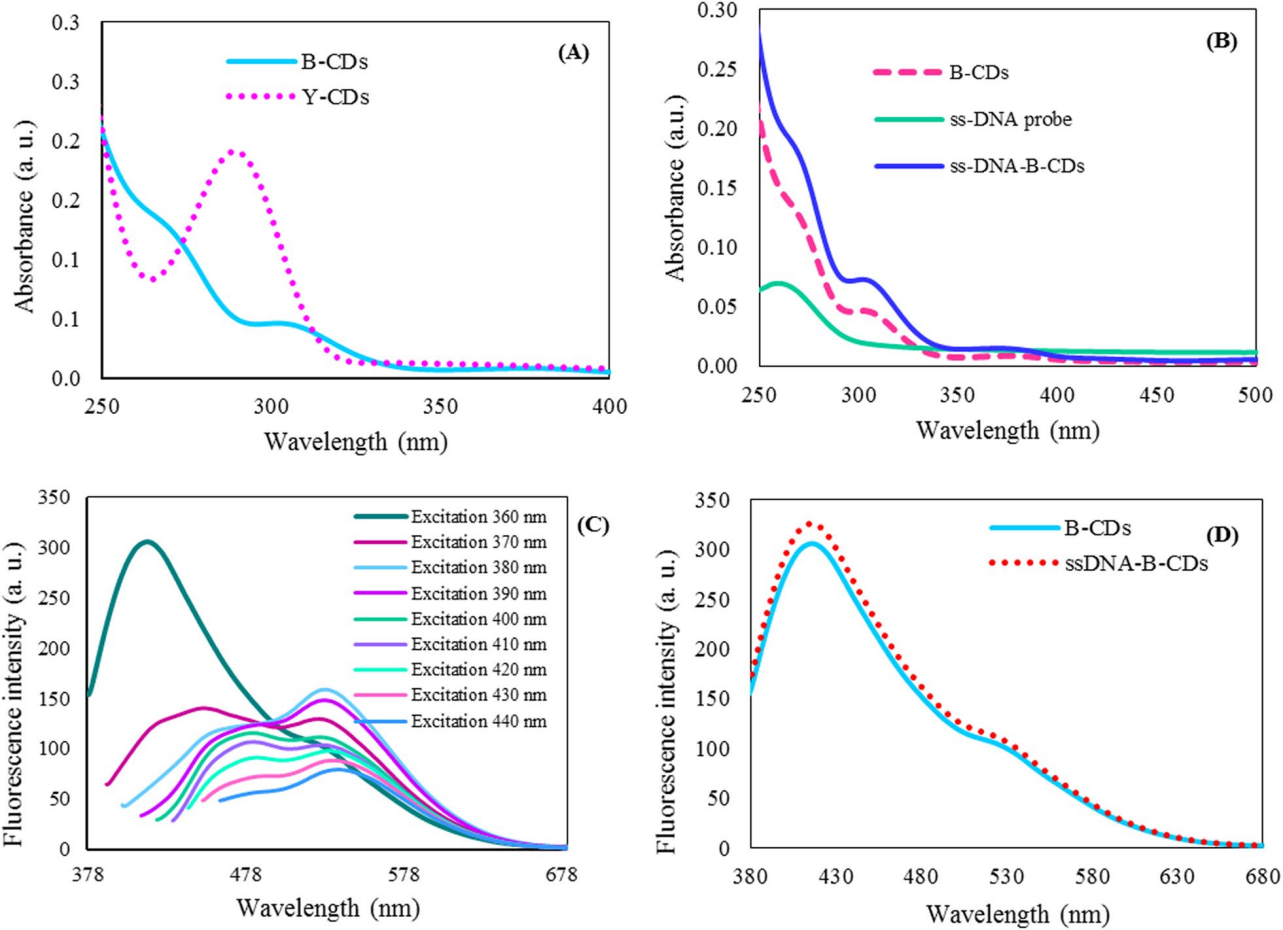
UV–vis absorption of B-CD and Y-CD (**A**); UV–vis absorption of B-CD, DNA probe and B-CD-DNA probe (**B**); Fluorescence spectra of B-CDs at different excitation wavelengths from 360 to 440 nm (**C**); Fluorescence spectra of B-CD and DNA probe functionalized B-CD (**D**)

The optical properties of B-CDs and Y-CDs and their mixtures were studied by a recording UV–vis absorption and fluorescence (FL) spectra. First, as predicted, all the spectra display two main absorbance peaks. One of the peaks is a strong absorption in the UV region, which is ascribed to the typical π–π* transition of the aromatic sp^2^ core domain. Moreover, the absorption peak at ≈ 300 nm is attributed to n–π* transition of surface functional groups with lone pairs electron to the π* orbital of the sp^2^ carbon domain (Fig. 2A) [44]. The absorbance spectra of B-CD, DNA probe, and B-CD-DNA probe attained in Fig. 2B propose the successful immobilization of DNA probe onto B-CD surface. The UV–vis absorption peaks were observed at about 270 and 305 nm for B-CD and 260 nm for DNA probe. The covalent interaction between B-CD and DNA probe led to increasing the peaks at around 270 and 305 nm that confirming the successful loading of DNA probe on B-CD. Moreover, the successful immobilization of the DNA probe on B-CD was verified by electrochemical impedance spectroscopy (EIS). As shown in Additional file 1: Fig. S2, the bare GCE displayed a small semicircle, while the resistance increased for the electrode immobilized with B-CD. For the BCD-DNA probe loaded on an electrode, a further increase in resistance was observed, which confirmed the B-CD modification with the DNA probe.

CDs usually possess the excitation wavelength-dependent emission, as presented in Fig. 2C, the prepared B-CDs reveal excitation wavelength-dependent emission behavior, displaying various emission wavelengths ranging from 415 to 485 nm and 520 to 540 nm at excitations from 360 to 440 nm. It seems that the excitation-dependent emission is originated from various fluorescence centers and complex energy levels of CDs, affecting their bandgaps [44, 45]. The mixture of B-CDs and Y-CDs (BY-CDs) showed a fluorescence spectrum with emission peaks at 410 nm and 540 nm upon excitation at 360 nm (Additional file 1: Fig. S3). The conjugated aromatic π structures and also hydrogen bonding among carboxylic acid, carbonyl functional groups, and –NH_2_, –OH on CDs may enhance the rigidity and lead to the shift of the absorption and emission wavelengths [46]. In the case of fluorescence emission, an increase in fluorescence signal of B-CDs is realized after DNA probe functionalization rooted in alterations in the steric and electronic features, likely created by the enhance of nitrogen moieties after modification with DNA probe in addition to enhancing its rigidity [47] (Fig. 2D).

### Optimization of experimental conditions

**Fig. 3:**
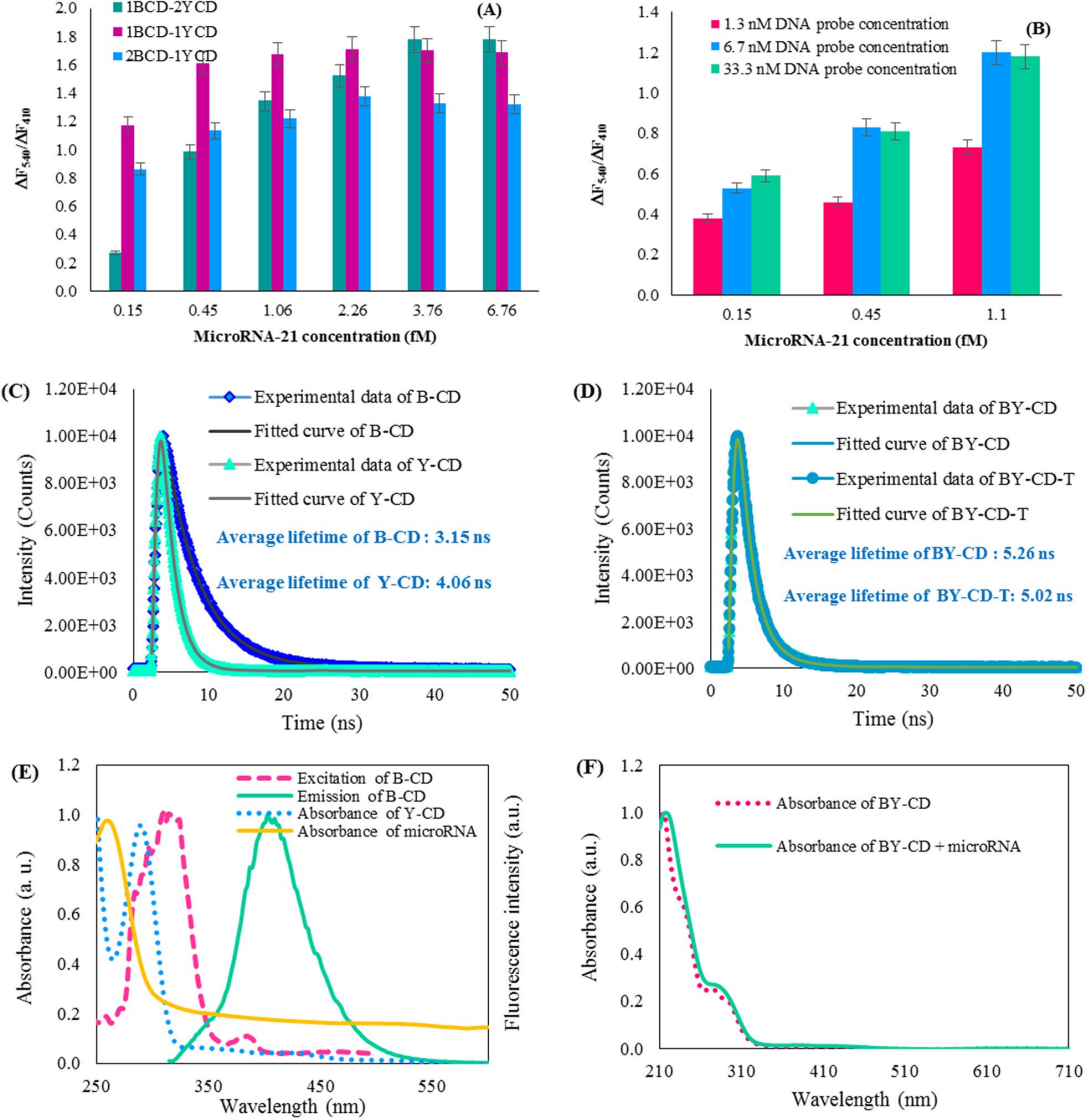
The effect of the various volume ratio of B-CDs and Y-CDs (**A**); The effect of DNA probe concentration to modification of B-CDs surface (**B**) for microRNA-21 detection; Fluorescent lifetime of B-CD and Y-CD (**C**); Fluorescent lifetime of BY-CD before and after adding microRNA (**D**); Absorbance spectra of Y-CD (blue), microRNA (yellow) and excitation (pink) and emission (green) fluorescence spectra of B-CD (**E**); Absorbance spectra of BY-CD before and after adding microRNA (**F**)

To improve the detection performance for target microRNA-21, the experimental conditions including B-CDs –Y-CDs ratio, DNA probe concentration, and incubation time were optimized using fluorescence intensity ratio defined as ΔF_540_/ΔF_410_. To construct the dual emission probe, B-CDs and Y-CDs were mixed in the different volume ratios of 2:1, 1:1, and 1:2 (B-CDs: Y-CDs), and various microRNA-21 concentrations in the range of 0.15–6.76 fM were added to the prepared solution. Upon excitation of 360 nm, blue-emitting CDs and yellowemitting CDs exhibited a maximum emission at 415 and 435 nm, respectively. The combination of two types of CDs led to a dual emission photoluminescence spectrum under a single wavelength excitation (360 nm) at 410/543 nm. The blue shifting the maximum emission wavelength ~ 410 nm may originate from increasing particle size [48]. As shown in Fig. 3A, the ΔF_540_/ΔF_410_ ratio was the best when using a 1:1 volume ratio of B-CDs and Y-CDs in the system.

DNA probe is one such material that has attracted wide attention owing to its physical features, various structural engineering abilities, low-cost synthesis procedure, and unparalleled customizability for exactly arranging target-binding sites at the nanostructures. These features make the DNA probe a superb option for the construction of biosensing methods [49]. Here, a DNA probe was exploited for functionalization of B-CDs as the microRNA-21 sensing probe to enable a great number of DNA probes to hybridize with target microRNA-21 with improved probe availability and high sensitivity. Moreover, the functionalization of DNA probes onto B-CDs surface was comprehended, via amidization method among -COOH groups on B-CDs and -NH_2_ modified DNA probes [50]. As shown in Fig. 2B and D, absorbance and fluorescence spectra exhibited the binding between DNA probe and B-CDs. As shown in Fig. 3B, the effect of various amounts of DNA probes (1.3, 6.7, and 33.3 nM) was examined for modification of B-CDs surface and the best fluorescence response was obtained for a concentration of 6.7 nM of DNA probes. Therefore, 6.7 nM was selected as the optimal amount of the DNA probe. Furthermore, the fact that the stability of the assay is a significant factor affecting the reproducibility and accuracy of fluorescence measurement is clear. Therefore, the stability of the aqueous solution of BY-CDs and upon addition of microRNA-21 to the BY-CDs system was investigated (Additional file 1: Fig. S4a–d). The fluorescence intensity ratios (F_540_/F_410_) were calculated throughout 60 min that the results revealed the signal remained almost constant. To guarantee a good interaction between probe and target, the fluorescence response was measured within a reaction time of 20 min.

### Analytical performance of the designed biosensor

Under optimal conditions, the sensing performance of BY-CDs assay for microRNA-21 detection was investigated. As shown in Fig. 2D, B-CDs displayed a maximum FL emission at 415 nm and a shoulder at 520 nm when excited at 360 nm. Upon addition of 15 µg mL^−1^ of Y-CDs to 15 µg mL^−1^ of DNA functionalized B-CDs, two fluorescence signals can be observed at 410 nm and 540 nm under 360 nm excitation. As can be seen in Fig. 3C, D, the average fluorescence lifetime of probe B-CDs before and after the addition of Y-CDs were 3.15 ns and 5.61 ns, respectively. Therefore, the fluorescence behavior change of the system can be interpreted as a result of the altered local environment by the interaction between B-CDs and Y-CDs. After the addition of microRNA-21, the fluorescence intensity decreased at 410 nm while the enhanced fluorescence at 540 nm can be detected. The sensing mechanism was investigated using absorbance, fluorescence, and lifetime decay experiments. As can be observed in Fig. 3E, the absorption spectrum of microRNA overlapped with the excitation spectrum of the B-CDs. Therefore, the possible fluorescence quenching mechanism can be owing to the inner filter effect (IFE). To verify the IFE, the average fluorescence lifetime of the fluorophore was measured in the absence and the presence of microRNAs (Fig. 3D). The values were calculated to be 5.26 and 5.02 ns, respectively. Since the average amount of lifetime remains almost unchanged, the mechanism can be the IFE. Furthermore, the absorption spectra of the BY-CDs before and after microRNA addition can be the best technique to discriminate between the IFE and static processes. Since the absorption spectrum of the BY-CDs would not alter (Fig. 3F), suggested that fluorescence quenching is because of the IFE rather than the static process [51, 52]. The specific binding of DNA probe functionalized B-CDs to complementary microRNA-21 target induced perturbations of probe structure and led to an increase of fluorescence intensity at 540 nm. The signal ratio of ΔF_540_/ΔF_410_ was applied as a final readout for microRNA-21 determination. The relationship between ΔF_540_/ΔF_410_ and logarithm microRNA-21 concentration is displayed in Fig. 3A. The value of ΔF_540_/ΔF_410_ linearly depended on the logarithm of the microRNA-21 concentration in the range of 0.15 fM to 2.46 pM with a correlation coefficient of 0.992. The detection limit for microRNA-21 was calculated to be 50 aM (S/N = 3). The obtained detection limit is much lower than or comparable to most of the previously reported methods (Table 1). Compared to other ratiometric techniques, not only is this probe extremely sensitive without a complex signal amplification method, but it also possesses the benefit of being affordable and environment-friendly.

**Table 1:** Nanomaterial-based ratiometric sensors for determination of microRNA

Detection technique	Micro/Nanomaterials	Target	Linear range	Detection limit	References
Electrochemical	Black phosphorus nanosheets	MicroRNA-3123	2 pM to 2 μM	0.3 pM	[20]
Electrochemical	Au NPs	MicroRNA-141	0.1 fM to 100 pM	11 aM	[53]
Electrochemical	Magnetic beads	MicroRNA-21	1 pM to 10 nM	1 pM	[56]
Electrochemiluminescence	Au NPs functionalized g-C_3_N_4_ NS nanohybrid (Au-g-C_3_N_4_ NH)	MicroRNA-21	1.0 fM to 1.0 nM	0.5 fM	[21]
Chemiluminescence	Graphene oxide quantum dots (GOQD)	MicroRNA-21	0.005 to 50 pM	1.7 fM	[22]
SERS	Silica@Au nanoflower (Si@AuNF)	MicroRNA-122	10 aM to 100 pM	7.75 aM	[23]
Ratiometric fluorescence	Boron doped g-C_3_N_4_ nanosheets (BCNNS) & copper nanocluster (CuNC)	MicroRNA-582-3p	0.2 to 1.0 pM	12.0 fM	[25]
Ratiometric fluorescence	Carbon dots	MicroRNA-21	0 fM to 500 fM	3 pM	[38]
Ratiometric fluorescence	Carbon dots	MicroRNA-21	50 to 10,000 pM	1 pM	[26]
Fluorescence resonance energy transfer (FRET)	Conjugated polymer (CCP) & fluorescein	let-7a family	0.2 to 100 pM	0.08 pM	[54]
FRET	Fluorescence dyes Cy3,Cy3.5, Cy5	MicroRNA-155, microRNA-182, and microRNA-197	0.02 to 10 nM	18 pM, 12 pM, and 11 pM	[55]
Ratiometric fluorescence	Carbon dots	MicroRNA-21	0.15 fM to 2.46 pM	50 aM	This work

**Fig. 4:**
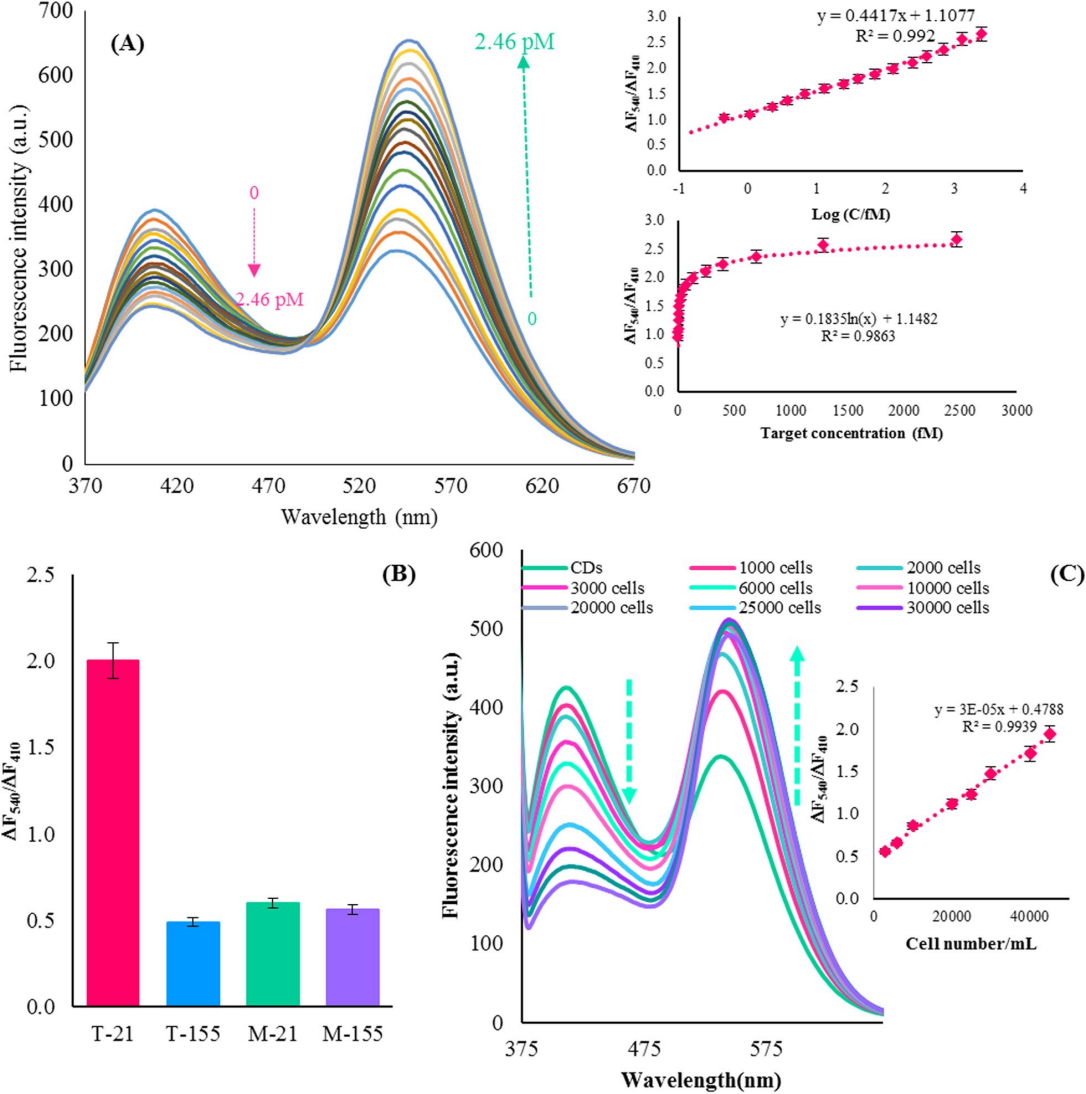
Fluorescent responses of the dual-emission BY-CD-based ratiometric assay at different concentrations of microRNA-21 from 0.15 fM to 2.46 pM, Inset: linear relationships of ΔF_540_/ΔF_410_ versus log C (microRNA-21) and logarithmic relationships of ΔF_540_/ΔF_410_ versus target concentration (microRNA-21) (**A**); Selectivity of the dual-emission BY-CD-based assay toward microRNA-21 sensing. The concentrations of microRNA-21 (T-21), and interferences, including microRNAs-155 (T-155), one base-mismatched microRNA-21 (M-21), and one base-mismatched microRNA-155 (M-155) were all 0.3 pM (**B)**; Fluorescent responses and the linear relationship between ΔF_540_/ΔF_410_ and the concentration of microRNA-21 extracted from of the MCF-7 cells from 3000 to 45,000 (cell mL^−1^) (**C**)

Sequence similarity among family members is a remarkable property of microRNA, and distinguishing dissimilarities among members of the microRNA family is a major challenge for microRNA determination. MicroRNAs-155 (T-155), one base-mismatched microRNA-21 (M-21), and one base-mismatched microRNA-155 (M-155) were employed to investigate the selectivity of this assay. As shown in Fig. 4B, a considerable change was recognized in the presence of microRNA-21, whereas other interfering microRNAs make a lower signal response than that of microRNA-21 at the same amount (0.3 pM). However, our consequences displayed that this system reveals high selectivity towards target microRNA-21 and can distinguish other microRNAs with analogous sequence and length.

### Application of the miRNA detection assay in serum and cancer cell line samples

To assess the practical application of the proposed system, target microRNA detected in cancer cells and serum samples. Usually, the concentration of microRNA in biological samples is not adequate to assess microRNA since circulating microRNAs are present at femtomolar concentrations or even lower levels in the blood samples [57]. The appropriate amounts of microRNA-21 (0.15 and 0.5 fM) were spiked into 1.0% (v/v) healthy human serum and quantified by the ratiometric fluorescence biosensor. Acceptable recovery rates (93.3–98.0%) and relative standard deviations (RSD %) (2.9–5.4%) were acquired for spiked serum samples (Table 2), representing the favorable potential of the presented ratiometric assay for detecting microRNA-21 in real human serum.

**Table 2:** Results from ratiometric nanobioensor for detection of microRNA-21 in serum samples

Sample	Measured (fM)	Spiked (fM)	Found (fM)	Recovery %	RSD (%, n = 3)
**1**	0.13	0.15	0.265	90.0	2.9
		0.50	0.62	98.0	5.4
**2**	0.16	0.15	0.305	96.7	5.1
		0.5	0.625	93	3.2

Further investigation of the ratiometric fluorescence nanoprobe in response to varying cellular components, such as MCF-7 cell lines, exhibited high selectivity to microRNA-21 (Fig. 4C). Moreover, the nanoprobe provided decreasing fluorescence intensity at 410 nm and increasing fluorescence intensity at 540 nm with an enhanced concentration of microRNA-21 in the cell extraction samples from the MCF-7. Figure 4C shows that the linear relationship between ΔF_540_/ΔF_410_ and the concentration of microRNA-21 extracted from the MCF-7 cells from 3000 to 45,000 (cell mL^−1^). The detection limit was calculated to be 300 cell mL^−1^ (3 cells in 10 μL of the injected sample) at S/N of 3. These results demonstrated that this ratiometric nanoprobe holds great potential for microRNA analysis in different tumor cells, which is of great significance in biomedical research and clinical diagnosis.

### MicroRNA-21 imaging in MCF-7 cells

**Fig. 5:**
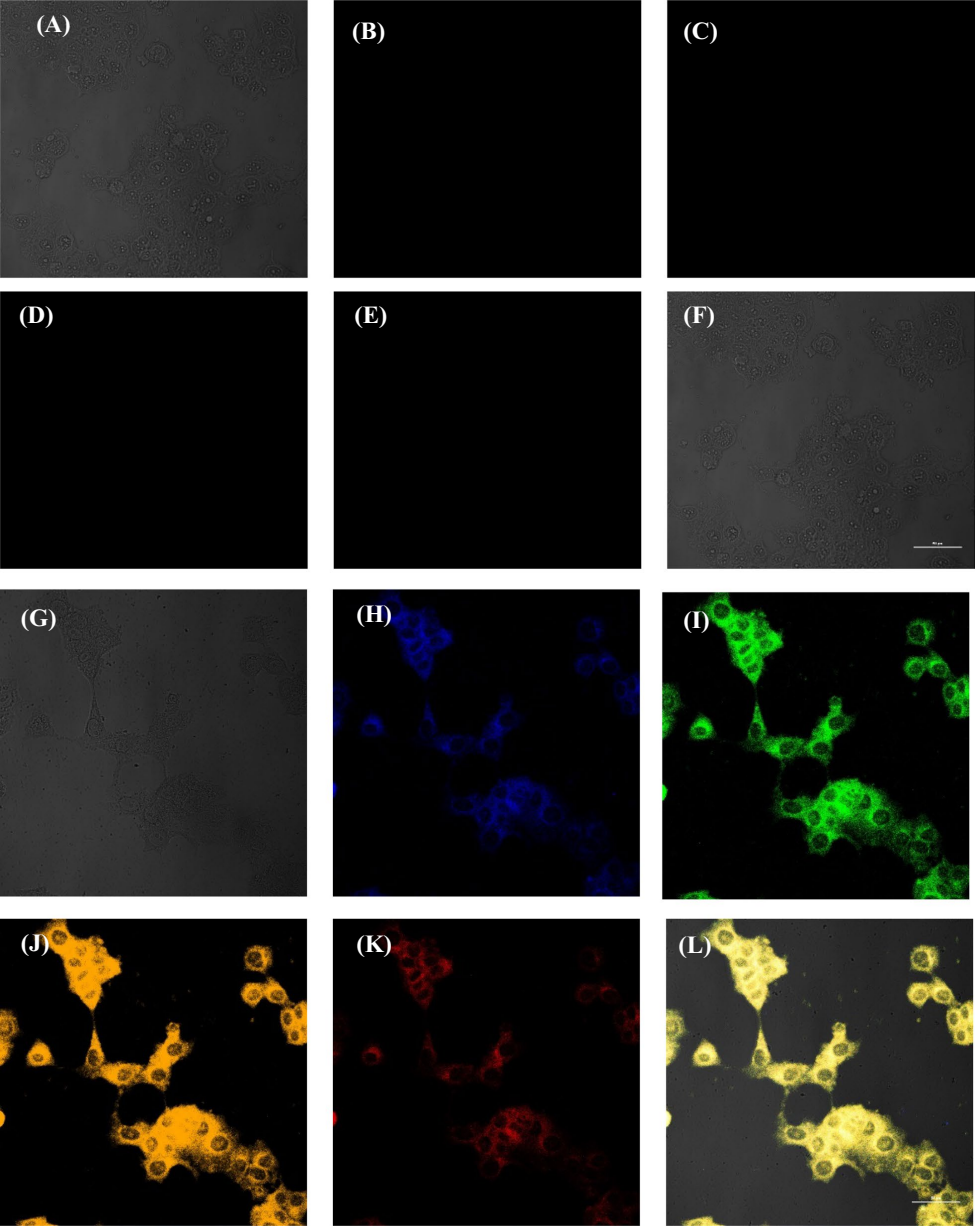
Confocal laser scanning microscopic images of MCF-7: control (**A**–**F**) cells and cells treated with 1.0 mg mL^−1^ of BY-CDs (**G**–**L**) excitation by bright field (**A**, **G**), Blue (**B**, **H**), Green (**C**, **I**, Yellow (**D**, **J**), Red (**E**, **K**) and Merge (**F**, **L**)

Dual-emissive CDs with outstanding water solubility and great optical features are anticipated to be a suitable fluorescent option for bioimaging. To investigate the bioimaging applications of CDs in MCF-7 cells, the relative viability of MCF-7 cancer cells exposed to BY-CDs was assessed by MTT assay at 545/630 nm. The results proved that the BY-CDs were not toxic to MCF-7 cells because more than 95% of the MCF-7 cells were viable when the concentration of the BY-CDs was less than 1.0 mg mL^−1^ (Additional file 1: Fig. S5). These MTT consequences reveal that modified BY-CDs can be exploited as non-toxic labels for cellular imaging usages. The reaction time of MCF-7 cancer cell imaging has been adjusted for 24 h. MCF-7 cancer cells treated with BY-CDs did not exhibit considerable changes in shape. As shown in Fig. 5A–L, robust blue, green, yellow, and red photoluminescence on MCF-7 cell membranes demonstrated that BY-CDs had moved in the cells and that fluorescence features had been preserved in the cellular medium, while no radiant light in the core is not visible. The images revealed that MCF-7 cells are well labeled with CDs and strong multicolor fluorescence could be proposed the applicability of BY-CDs in bioimaging.

### Applications in real-sample analysis on microfluidics

**Fig. 6:**
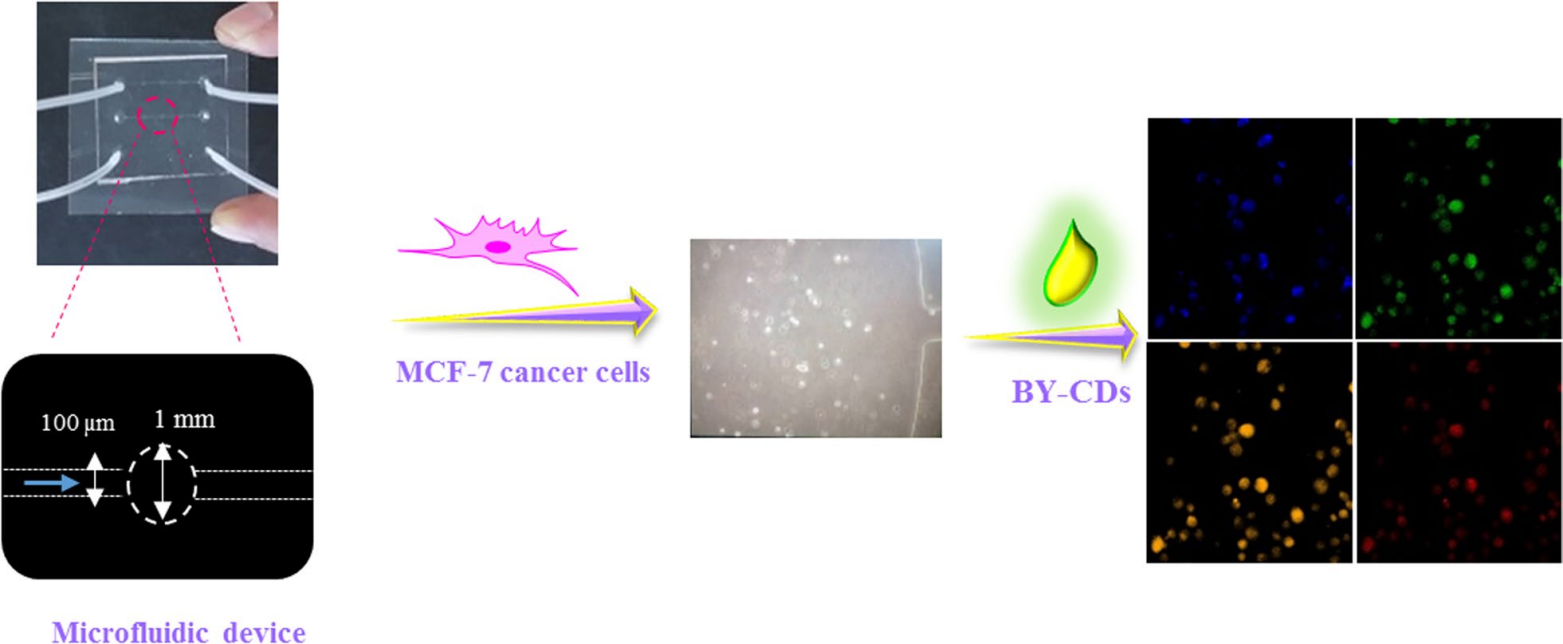
Confocal laser scanning microscopic images of MCF-7 cells treated with 0.5 mg mL^−1^ of BY-CDs in microfluidic device

Profited from the small sample volumes, ease of incorporation, fast analyses time, and high movability presented by microfluidics, microfluidic chips are one of the most remarkable technologies, which have been combined with fluorescence biosensor methods to increase their efficiency [58]. To increase the user accessibility, the MCF-7 cells were applied in a microfluidic device to allow imaging under a confocal fluorescence microscope. MCF-7 cells were introduced into the channels at a flow rate of 10 μL min^−1^ to assess the robustness of our procedures. After 24 h incubation at 37 °C, BY-CDs solution at a concentration of 0.5 mg mL^−1^ (dissolved in DMEM) was inserted into the microfluidic. After 5 h, the cancer cells were washed with PBS then fluorescence image analysis was attained under various excitation filters. As can be seen in Fig. 6, the MCF-7 cells answered to the full spectrum of excitation light by emitting signals. Furthermore, the efficiency of the biosensor in the microfluidic chip was preserved. The high portability, speed of analysis, and low sample consumption make this tool an ideal format for rapid point-of-care diagnosis methods.

## Conclusions

In summary, a highly sensitive ratiometric fluorescence assay was developed for microRNA-21 detection at aM concentration range in serum samples and also in MCF cell lines using blue and yellow carbon dots (B-CDs, Y-CDs, and F_540nm_/F_410nm_) as fluorophores. The proposed fluorescent nano biosensor has become a robust approach in analytical sensing owing to its rapid response, great sensitivity, and technical simplicity. Furthermore, due to the low toxicity of CDs, we constructed a microfluidic device for fluorescent imaging of microRNA in MCF-7 cells using DNA probe conjugated dual emissive CDs. Not only did this nanoprobe provide a highly sensitive procedure for microRNA-21 detection, but it also enabled to distinguish of single-base mismatched microRNAs. Moreover, this procedure effectively evaluated for quantification of microRNA-21 in clinical serum samples. The acceptable recovery and reproducibility results demonstrated that the bioassay provides the possibility of a diagnosis of microRNA-related diseases. Although this assay was utilized for single-analyte detection, our study suggested the possibility of developing sensitive, robust biosensors for the determination of multiple analytes to meet clinical needs in disease-related microRNA.

## Supplementary Information

**Additional file 1.** Additional informations includes SEM images of CDs, impedance data, UV–vis and fluorescence spectra of B-CD and Y-CD mixture and Cellular cytotoxicity evaluation of the BY-CD toward MCF-7 cells.

## Data Availability

All data analyzed during this study are included in this published article.
