# Supplementary material for: Carbon dots hybrid for dual fluorescent detection of microRNA-21 integrated bioimaging of MCF-7 using a microfluidic platform
Source: J Nanobiotechnology. 2022 Feb 8;20:73. doi: 10.1186/s12951-022-01274-3 (PMC8822830; doi:10.1186/s12951-022-01274-3)
Supplement: Supplementary file 1 — Additional file 1. Additional informations includes SEM images of CDs, impedance data, UV–vis and fluorescence spectra of B-CD and Y-CD mixture and Cellular cytotoxicity evaluation of the BY-CD toward MCF-7 cells. [file 12951_2022_1274_MOESM1_ESM.docx]

**Supporting file**

**Carbon dots hybrid for dual fluorescent detection of microRNA-21 integrated bioimaging of MCF-7 using a microfluidic platform**

Somayeh Mohammadi^1^, Abdollah Salimi,^1,2^* Zohreh Hoseinkhani^3^, Foad Ghasemi^4^, Kamran Mansouri^3^

^1^ Department of Chemistry, University of Kurdistan, Sanandaj 66177-15175, Iran

^2^ Research Center for Nanotechnology, University of Kurdistan, Sanandaj 66177-15175, Iran.

^3^ Medical Biology Research Center, Kermanshah University of Medical Sciences, Kermanshah, Iran

^4^Nanoscale Physics Device Lab (NPDL), Department of Physics, University of Kurdistan, Sanandaj, 66177-15275, Iran.

Corresponding author

* Abdollah Salimi

Phone: +98-87 33624001, e-mail: [absalimi@uok.ac.ir](mailto:absalimi@uok.ac.ir);

**
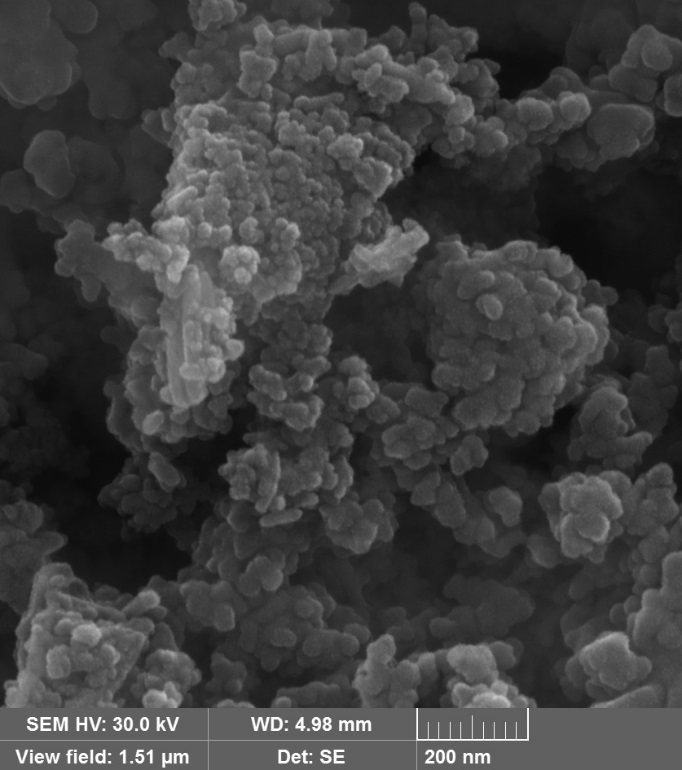
**

**Figure S1.** SEM image of the CDs;

**Figure S2.** Nyquist plots of GC electrode, GCE/B-CD and GCE /B-CD-DNA probe in phosphate buffer (pH 7.4) containing 2.5 mM [Fe(CN)6]3-/4- and 0.1 M KCl, applied potential was 0.2V at the frequency range of 0.1Hz-10kHz

**Figure S3.** UV-vis absorption and fluorescence spectra of B-CD and Y-CD mixture


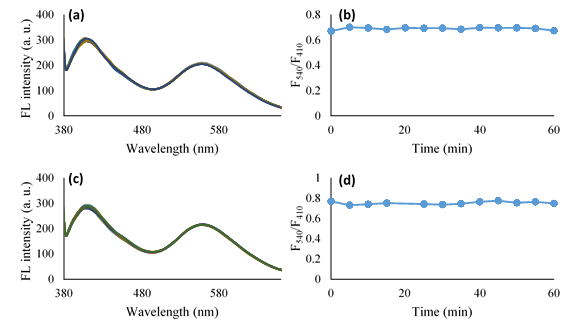


**Figure S4.** Fluorescence spectra of BY-CDs (a) BY-CDs ratiometric assay upon the addition of o.15 fM microRNA-21 (c) and the effect of reaction time on fluorescence intensity ratio (F_540_/F_410_) of BY-CDs (b), BY-CDs ratiometric assay upon the addition of o.15 fM microRNA-21 (d)

**Figure S5.** Cellular cytotoxicity evaluation of the BY-CD toward MCF-7 cells
